# Assessing regression to the mean effects in health care initiatives

**DOI:** 10.1186/1471-2288-13-119

**Published:** 2013-09-28

**Authors:** Ariel Linden

**Affiliations:** 1 Linden Consulting Group, LCC Ann Arbor, MI, USA; 2 Department of Health Management & Policy, School of Public Health, University of Michigan, Ann Arbor Michigan, USA

**Keywords:** Regression to the mean, Outliers, Validity, Outcomes, Confidence intervals, Simulation

## Abstract

**Background:**

Interventions targeting individuals classified as “high-risk” have become common-place in health care. High-risk may represent outlier values on utilization, cost, or clinical measures. Typically, such individuals are invited to participate in an intervention intended to reduce their level of risk, and after a period of time, a follow-up measurement is taken. However, individuals initially identified by their outlier values will likely have lower values on re-measurement in the absence of an intervention. This statistical phenomenon is known as “regression to the mean” (RTM) and often leads to an inaccurate conclusion that the intervention caused the effect. Concerns about RTM are rarely raised in connection with most health care interventions, and it is uncommon to find evaluators who estimate its effect. This may be due to lack of awareness, cognitive biases that may cause people to systematically misinterpret RTM effects by creating (erroneous) explanations to account for it, or by design.

**Methods:**

In this paper, the author fully describes the RTM phenomenon, and tests the accuracy of the traditional approach in calculating RTM assuming normality, using normally distributed data from a Monte Carlo simulation and skewed data from a control group in a pre-post evaluation of a health intervention. Confidence intervals are generated around the traditional RTM calculation to provide more insight into the potential magnitude of the bias introduced by RTM. Finally, suggestions are offered for designing interventions and evaluations to mitigate the effects of RTM.

**Results:**

On multivariate normal data, the calculated RTM estimates are identical to true estimates. As expected, when using skewed data the calculated method underestimated the true RTM effect. Confidence intervals provide helpful guidance on the magnitude of the RTM effect.

**Conclusion:**

Decision-makers should always consider RTM to be a viable explanation of the observed change in an outcome in a pre-post study, and evaluators of health care initiatives should always take the appropriate steps to estimate the magnitude of the effect and control for it when possible. Regardless of the cause, failure to address RTM may result in wasteful pursuit of ineffective interventions, both at the organizational level and at the policy level.

## Background

Interventions targeting individuals classified as “high-risk” have become common-place in the health care industry. High-risk may capture anything from high utilization or cost of health services, to outlier values on clinical measures (e.g., blood glucose, blood pressure, cholesterol). Typically, such individuals are invited to participate in an intervention intended to reduce their level of risk, and after a period of time, a follow-up measurement is taken. The pre-test to post-test change in the outcome is then generally presented as the impact of the intervention. This evaluation approach is problematic from a statistical standpoint because individuals initially identified by their high values will likely have lower values on re-measurement in the absence of an intervention. This statistical phenomenon is known as “regression to the mean” (RTM) and often leads to an inaccurate conclusion that the intervention resulted in a treatment effect [1].

The implications of RTM in evaluating medical interventions have been examined extensively in the literature, suggesting that RTM is a common problem [2-21]. However, RTM is rarely addressed when evaluating health care delivery interventions or in the more general decision making processes in health care [22]. This is despite its increasing relevance given the intensified focus on high-cost, and high-need groups, and efforts to design programs specifically targeting them. There are at least three possible explanations for why this may be. First, evaluations of delivery-side interventions are traditionally not subject to the same rigor as medical interventions (i.e., RCTs), and with this may come a lack of awareness of the need to address RTM. Second, it has been shown that cognitive biases may cause people to subconsciously systematically misinterpret RTM effects as intervention effects by creating (erroneous) explanations to account for it [23]. Third, there are more blatant examples in which organizations have a stake in the outcome of the intervention and capitalize on the RTM effect as a business strategy. For example, commercial disease management organizations have long advocated that their programs be evaluated without a control group despite the recognition that the intervention group will demonstrate better outcomes due to regression to the mean [24]. Regardless of the cause, failure to address RTM may result in wasteful pursuit of ineffective interventions, both at the organizational level and at the policy level.

In this paper, we seek to provide researchers, organizational decision-makers, and policy-makers, with a broa der set of tools to understand and assess RTM effects. First, real examples of RTM in health care are presented to illustrate the phenomenon. Next, the traditional method for calculating the RTM effect in normally distributed data is described, and these RTM effect estimates are compared with RTM effects generated from Monte Carlo simulation of normally distributed data. Next, these comparisons are repeated using skewed data from a control group in a health coaching study to illustrate the shortcomings of the traditional approach to accurately estimate the RTM effect, in the common scenario of non-normal data. We use this to motivate the primary contribution of the paper, the estimation of standard errors and confidence intervals around the RTM effect. While largely absent from existing RTM literature, including confidence intervals, a measure of the precision of single-value RTM estimates, is valuable because it provides a range of values that are considered to be plausible for the population. Finally, the advantage of calculating confidence intervals around RTM estimates is discussed in detail, and approaches for designing health care interventions to mitigate, or at least account for, the effects of RTM are provided.

## Methods

### The regression to the mean concept

Regression to the mean was first described over a century ago by Francis Galton (later Sir Francis) upon discovering that, on average, tall parents have children shorter than themselves and short parents have taller children than themselves [25]. RTM is the result of both random measurement error and extremity of scores from the mean [26]. A simple example of this occurs in measuring blood pressure or heart rate. Rarely are any two observations identical, even if taken minutes apart, due to natural biologic variability or measurement error. At the individual level this is called within-subject variability. Additionally, the more extreme the initial value, the greater the expected change will be in the follow-up score. However, over the course of many repeated observations, this variability narrows around the true mean [27,28]. Similar to individual level measures, groups with high (or low) initial mean values will also tend to regress to the mean of the overall sample.

In the context of an intervention, RTM can easily be mistaken for a program effect in the absence of an equivalent comparison group. The best approaches to illustrate the RTM phenomenon are either by using observations taken from time periods in which no interventions were implemented, or by using control group data derived from a research study.

**Figure 1:**
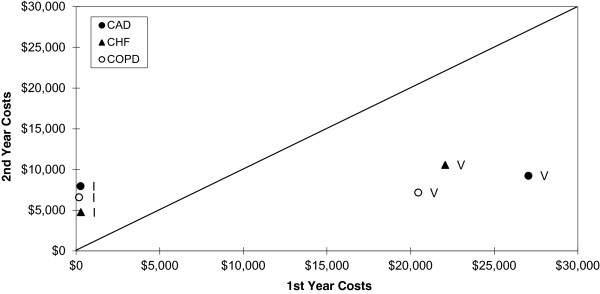
**Actual data illustrating the regression to the mean phenomenon in Coronary Artery Disease (CAD), Congestive Heart Failure (CHF), and Chronic Obstructive Pulmonary Disease (COPD).** Quintile I is the lowest cost group and V the highest. All individuals were continuously enrolled during the 2-year period. The diagonal line represents perfect correlation between the first and second year costs, which can only be achieved in the complete absence of variability between measurements and no measurement error.

Figure 1 illustrates the first approach by displaying the average costs for the highest and lowest quintile of a continuously enrolled cohort of chronically ill health plan members over the course of two years during which no chronic disease interventions were in place [29]. Each cohort - coronary artery disease (CAD), congestive heart failure (CHF), and chronic obstructive pulmonary disease (COPD) - exhibits a similar RTM pattern. In the first year, the highest quintile average costs range from approximately $20,000 to $27,000 across the three conditions. In the second year, the average costs in these groups drop to a range of approximately $7,000 to $10,000. Conversely, all three cohorts in the lowest quintile of costs (less than $300) in the first year increased to between $4,700 and $8,000 in the second year. The diagonal line is the expected trend line had there been perfect correlation between the first and second measurements (i.e., no variability between measurements, no measurement error, and thus no RTM). This scenario clearly illustrates RTM. Had a disease management program targeting high-cost CAD, CHF, or COPD patients taken place during this period, an evaluation of the impact on costs would have wrongly attributed these reductions to a program effect.

**Figure 2:**
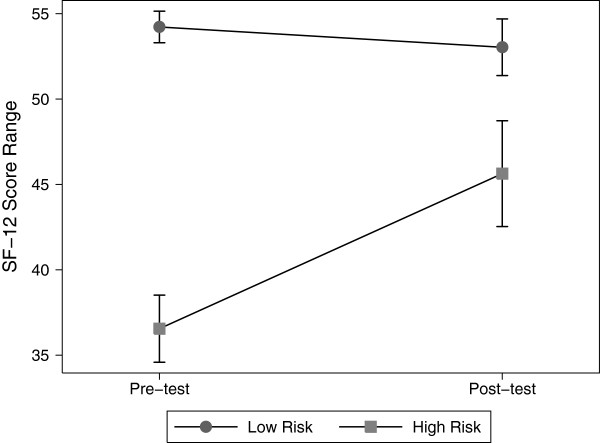
**Physical Component Summary (PCS) scores on the Short Form-12 (SF-12v2), from a control group (*n *= 118) participating in a health coaching study (Butterworth et al. 2006).** All participants were surveyed twice, once at program commencement and then again at three months. Squares/circles represent mean scores and capped lines represent 95% bootstrapped confidence intervals (1000 resamples).

Using data from a control group also illustrates RTM. Figure 2 presents physical component summary (PCS) scores (with bootstrapped 95% confidence intervals) from the SF-12 health status survey [30] for a control group (*n* = 118) from a study conducted at a large organization in the Northwest [31]. Control group members were surveyed twice, once at program commencement and then again at three months and received no intervention. Scale values are standardized from 0 to 100, with higher values indicating better physical health. To illustrate RTM, if a high-risk group is classified as having a PCS score in the first period of less than 44.25, which corresponds to the 25^th^ percentile at the U.S. national level [32], RTM is evident in the fact that their mean PCS scores significantly increases (no overlap in the pre- and post-measurement confidence intervals), by over 8 points (22.6%), in the second period while the lower-risk group (those in the 26^th^-100^th^ percentile) remained unchanged (because the mean value of this group was already close to the overall mean of the entire sample, there was nowhere to regress to).

The examples presented in this section use measures that commonly serve as outcomes in health care interventions, and both cases clearly illustrate RTM. This suggests that there are likely many contexts in which RTM, and not a program effect, explains an observed change from initial outlier status to follow-up values closer to the overall mean.

### Classic formulae for estimating the magnitude of RTM

Estimation of the RTM effect for normally distributed data can be conducted when, at a minimum, the following four parameters are known: the population mean of the pre-test (μ), the population variance of the pre-test (σ^2^), the correlation between the pre-test and post-test (ρ), and the cutoff score representing the high-risk group (κ). The expected RTM effect is [27,33,34]:

(1) $$\text{Expected}\;\mathrm{RTM}\;\text{effect}=\frac{\gamma^{2}}{\sqrt{\left(\gamma^{2}+\delta^{2}\right)}}C\left(z\right)$$

where *y*^*2*^ is the within-subject variance (*σ*^2^ − *δ*^2^), *δ*^2^ is the between-subject variance (*ρσ*^*2*^), and thus (*δ*^2^ + *γ*^2^) is the pooled variance (when the square root is taken, this becomes the pooled standard deviation). *C(z)* is calculated iteratively, beginning with the *z*-score:

(2) $$z=\left(\kappa -\mu \right)/\sigma$$

whenever high-risk is indicated by values above κ, and

(3) $$z=\left(\mu -\kappa \right)/\sigma$$

whenever high-risk is indicated by values below κ. As before, μ is the baseline population mean, σ is the standard deviation of the entire pre-test sample, and κ is the cutoff score. *C* is calculated as:

(4) $$C=\phi \left(z\right)/\left(\left[1-\Phi \left(z\right)\right]\right)$$

where *Φ(z)* is the probability density function and *Φ(z)* is the cumulative distribution function for z in a standard normal distribution.

The expected mean values for both pre-test and post-test in the high-risk group can also be calculated as follows:

(5) $$\text{Expected}\;\text{pre}-\text{test}\;\text{mean}\;\left(\text{high}-\text{risk}\right)=\mu \pm \mathrm{C\sigma}$$

(6) $$\text{Expected}\;\text{post}-\text{test}\;\text{mean}\;\left(\text{high}-\text{risk}\right)=\mu \pm \mathrm{C\sigma \rho}$$

where values are added whenever high-risk is indicated by values above κ, and subtracted whenever high-risk is indicated by values below κ. Subtracting the expected pre-test mean (Equation 5) from the expected post-test mean (Equation 6) should elicit the same expected RTM effect as that derived in Equation 1 (as will *Cσ(1-ρ)*).

### Testing the performance of the RTM formulae

We examine the performance of the RTM equation (Equation 1) in estimating the RTM effect using two approaches. First a Monte Carlo simulation study is conducted assuming medical cost as the outcome, as it is often a primary focus of health services research. Following the simulation, the performance of the RTM formulae is demonstrated using actual data (the PCS data described in the previous section).

#### *Design of the Monte Carlo simulation*

An “actual” RTM effect is generated by drawing two variables from a multivariate normal distribution to represent the pre-test and post-test costs for a pseudo-population of 10,000 observations, with means of $5,000, standard deviations of $1,350, and three pretest-posttest correlations: 0.25, 0.50 and 0.75. The minimum value of the highest pre-test quintile of cost is set as the cutoff (≈ $6,136), with values above and below that level categorized as “high-risk” and “low-risk”, respectively. The mean difference in pretest-posttest costs for the two risk tiers represents the “actual” RTM effects. We compare this with the “calculated” RTM effect for the high- and low-risk groups using Equation 1 with the same cutoff value (≈ $6,136). This process is repeated 10,000 times for each of the three correlation levels and the actual versus calculated RTM effects are reported for the low and high-risk groups. The simulation was conducted in Stata 12.1 (StataCorp, College Station, TX), using the built in *simulate* command, and *rtmci*, a command written by the author (available upon request).

#### *Design of the empirical example*

Here, the PCS data for the 118 controls [31] described earlier in the current paper and illustrated in Figure 1 are revisited, in order to demonstrate the performance of the RTM formulae when data are skewed (*p*<0.00001 for the Shapiro-Wilk W test). The pre-test mean, post-test mean, and mean difference in pretest-posttest PCS scores are used to generate the “actual” RTM effects and the “calculated” comparisons are again computed using Equations 1, 5 and 6 and *rtmci* in Stata. The differences between the actual and calculated values are then compared, and for all estimates, 95% confidence intervals are computed via bootstrap simulation, e.g., by resampling 1000 observations (with replacement) from the actual data.

## Results

### Monte Carlo simulation

Table 1 presents the simulation results. For each correlation level (0.25, 0.50, and 0.75), we report the actual and calculated mean RTM effects for the high- and low-risk groups, the standard errors, and 95% confidence intervals. As expected, the RTM effects diminish as the correlation between pre-test and post-test increases. When *ρ* = 0.25, the actual RTM effect in the high-risk group is $1,417. That is, there is a $1,417 mean decrease in the pre-test to post-test costs for the high-risk group that is entirely due to regression to the mean. The RTM effect decreases to $472 when *ρ* = 0.75. Thus, this simulation validates the conceptual underpinnings of RTM and supports the findings presented in Figures 1 and 2.

**Table 1:** **Results of the Monte Carlo simulation (*N *= 10,000)**

	**Mean**	**Std. error**	**[95% Confidence Interval]**	
*ρ = 0.25*				
RTM (H) actual	1417.67	0.33	1417.03	1418.32
RTM (H) calculated	1417.55	0.20	1417.16	1417.94
RTM (L) actual	354.16	0.18	353.80	354.51
RTM (L) calculated	354.27	0.06	354.15	354.39
*ρ = 0.50*				
RTM (H) actual	945.21	0.28	944.66	945.76
RTM (H) calculated	945.10	0.14	944.82	945.38
RTM (L) actual	236.09	0.15	235.80	236.38
RTM (L) calculated	236.20	0.04	236.12	236.28
*ρ = 0.75*				
RTM (H) actual	472.71	0.21	472.30	473.11
RTM (H) calculated	472.59	0.08	472.44	472.74
RTM (L) actual	118.03	0.11	117.83	118.24
RTM (L) calculated	118.11	0.02	118.07	118.15

Notes: RTM (H) is the regression to the mean effect for the high-risk group, and RTM (L) is the regression to the mean effect for the low-risk group. “Actual” represents the RTM effect derived directly from the data, and “calculated” is derived using Equation 1. *ρ* is the pretest-posttest correlation for the entire sample.

### Empirical data

The summary statistics of the PCS data are as follows: pre-test overall sample mean = 53.12, pre-test overall sample standard deviation = 8.27, pretest-posttest correlation = 0.742, and the cutoff = 44.25. Table 2 provides results for the high-risk group (PCS values ≤ 44.25, *n*=34) As shown, the *actual* pre-test mean is 3.38 points lower than that derived by the calculated method and the *actual* post-test mean is 1.52 points higher than that derived by the calculated method. As a result, the actual RTM effect is 8.28 points, which is much higher than the calculated method that produces a point estimate of 3.38. The difference between these two estimates is 4.90 points with a confidence interval of 1.12 to 8.68 points.

**Table 2:** **Regression to the mean effects for Physical Component Summary (PCS) scores on the Short Form-12 (SF-12v2), from the high-risk (PCS values ≤ 44.25) subgroup of controls (*n *= 34) participating in a health coaching study (Butterworth et al. 2006)**

**Variable**	**Mean**	**Std. error**	**[95% Confidence Interval]**	
*Actual*				
Pre-test	36.65	2.21	32.32	40.97
Post-test	44.93	3.27	38.51	51.34
RTM	8.28	2.01	4.35	12.21
*Calculated*				
Pre-test	40.02	0.78	38.49	41.56
Post-test	43.41	1.52	40.44	46.38
RTM	3.38	0.94	1.54	5.22
Difference (Actual – Calculated)				
Pre-test	−3.38	0.83	−5.01	−1.74
Post-test	1.52	1.82	−2.04	5.09
RTM	4.90	1.93	1.12	8.68

Notes: “Actual” indicates that the pre-test, post-test and RTM effects were estimated directly from the data. “Calculated” indicates that Equations 1, 5 and 6 were applied directly to the existing data. Standard errors and 95% confidence intervals were derived by bootstrap resampling of the data 1000 times.

## Discussion

The results of the simulation study demonstrate that the formulae for estimating RTM effects [27,33] accurately calculate RTM when the data are normally distributed. By extension, these results support the use of RTM analysis in pre-post observational studies as a means of estimating the RTM effect. However the results using these skewed data, suggests the RTM calculation significantly under-estimated the true RTM effect by between 1.12 and 8.68 points. Generally, when researchers seek to calculate the RTM effect using skewed data, transforming the data to make them normally distributed before using the traditional formulae may suffice. However, if transforming data to another scale may lead to a loss of interpretability (as would be the case with SF-12 data), performing the calculations on the original scale and calculating confidence intervals that reveal the magnitude of the error offers an alternative approach that may be more useful. In our example, the confidence interval for the calculated RTM effect was 1.54 to 5.22, which overlaps with the actual RTM confidence interval of 4.35 to 12.21. Thus, the confidence interval for the calculated RTM effect provides a range of values more closely aligned with the true effect than the point estimate alone. A third option is to consider models devised to estimate regression to the mean effects in non-normally distributed data [35,36]. However, some of these approaches rely on non-parametric modeling approaches, such as kernel density estimators [36], and are sensitive to the choice of bandwidth. Thus, the various approaches to estimating RTM may likely elicit different estimates depending on which methods are employed, even within the same data-set. Here again, the addition of confidence intervals can provide assistance to the evaluator in determining the overlap in estimates derived among the various methods.

### Designing interventions to mitigate the RTM effect

While earlier sections focused on illustrating RTM and offering suggestions for how to estimate the magnitude of the RTM effect, ideally studies are designed to mitigate the effect of RTM. The randomized-controlled trial (RCT) is the obvious study design to control for RTM because randomly assigned groups should be equally affected (i.e., the treatment effect is the net effect after eliminating any RTM). The regression-discontinuity (RD) design should be considered as a viable alternative when randomization is not possible [37,38]. The RD design relies on a cut-off point on a continuous pre-intervention variable to assign individuals to treatment. The individuals just to the right and left of the cutoff are assumed to be exchangeable - as in a randomized trial. Because individuals do not have precise control over their assignment score (nor would they know where the cutoff lies), they cannot self-select into treatment. Thus, we would expect a similar RTM effect for both groups in the neighborhood of the cutoff.

A third approach to mitigating the RTM effect in the design stage of an intervention is to base the treatment assignment on the cut-off, *conditioned on the mean of multiple pre-tests* rather than just a single pre-test [27,33,39,40]. This has the effect of stabilizing the mean and reducing within-subject variability. When multiple pre-test measurements are used, the previously described equations require minor modification [27,28,33]. In Equation 1, the within-subject variability *y*^*2*^ (in both the numerator and denominator) is now divided by the number of pre-tests *n* from which the mean is derived, becoming *y*^*2*^*/n*. In all other equations, σ is now replaced with the pooled standard deviation adjusted for multiple pre-test periods $\sqrt{\left(\gamma^{2}/n+\delta^{2}\right)}$.

### Controlling for RTM through data analysis

When only retrospective observational data are available, several approaches may be considered to control for RTM. Matching techniques [41] allow the investigator to try to replicate the randomization process by creating a control group that is essentially equivalent to the treatment group on observed pre-intervention characteristics – especially on the pre-test variable that we are most concerned leads to RTM. One particular advantage of matching techniques over other covariate adjustment strategies (e.g., multiple regression models), is that the investigator can directly assess how well the pre-test variable overlaps in its distribution between groups using graphical or numerical diagnostics [41]. A high degree of overlap in the distribution increases our confidence that the RTM is effectively controlled for, as we would expect in an RCT.

The most common analytic approach appearing in the literature to adjust for RTM is by analysis of covariance (ANCOVA). This approach controls for the baseline level of the pre-test by including the pre-test as a covariate in the model. Additionally, an RTM “correction factor” [42-44] can be applied to each person’s pre-test score and that adjusted pre-test score can be used in the ANCOVA. For example, Trochim [44] adjusts an individual’s pre-test score as follows:

(7) $$x_{\mathrm{adj}}=\bar{x}+p\left(x-\bar{x}\right)$$

where $\bar{x}$ is the treatment group mean, *ρ* is the pre-post correlation for that treatment group, and *x* is the individual’s pre-test value. It is important to keep in mind, however, that when using ANCOVA (with or without the corrected pre-test), model assumptions, such as linearity between outcome and covariates, must be tested. Moreover, contrary to matching strategies where covariate balance can be directly assessed, in ANCOVA models, there is no assurance that the treatment groups are comparable on all baseline covariates. In fact, it is imperative that decision-makers consider other potential sources of bias (in addition to RTM) that may masquerade as a treatment effect. This is particularly true when using observational data, since it is likely that participants and non-participants will differ on several characteristics (e.g., health behaviors) not often available in claims analysis [45].

Finally, perhaps the easiest approach for adjusting outcomes to control for RTM effects is simply to subtract the calculated RTM effect derived from Equation 5 from the overall treatment effect estimate [34]. Moreover, with the additional availability of confidence intervals, the investigator can provide a range of “net” treatment effect estimates when data are skewed.

## Conclusion

In this paper we have illustrated that health care interventions are susceptible to the effects of RTM when individuals are chosen to participate in the intervention based on their outlier baseline “risk” score, and there is large within-subject variability or measurement error. When estimating the RTM effect on normally distributed data the calculated estimates produce identical results to those of simulated data. However, the equations underestimated the RTM effect in right-skewed data. We described several approaches for investigators to consider as methods to adjust for RTM, depending on the degree of control they have over the intervention and evaluation designs. However, designing interventions to mitigate the effects of RTM is a preferred strategy to retrospectively estimating the extent to which RTM may explain any observed treatment effect. Most importantly, both evaluators and stakeholders should be aware of RTM as a major source of bias in intervention studies, and take the appropriate steps to estimating its effect and controlling for it whenever possible to ensure valid conclusions about program effectiveness.

## Competing interests

The author declares no competing interests.

## Authors’ contributions

The author designed the study, performed all statistical analyses and drafted the manuscript.

## Authors’ note

All of the proposed methods in this paper can be implemented via companion software, for Stata, which was written by the author and is available upon request.

## Pre-publication history

The pre-publication history for this paper can be accessed here:

http://www.biomedcentral.com/1471-2288/13/119/prepub

## References

[B1] Bland JM Altman DG Regression towards the mean BMJ 1994 308 1499 10.1136/bmj.308.6942.1499 8019287 PMC2540330

[B2] Boissel JP Duperat B Leizorowicz A The phenomenon of regression to the mean and clinical investigation of blood cholesterol lowering drugs Eur J Clin Pharmacol 1980 17 227 230 10.1007/BF00561905 6988223

[B3] Andrews G Harvey R Regression to the mean in pretreatment measures of stuttering J Speech Hear Disord 1981 46 204 207 7253599 10.1044/jshd.4602.204

[B4] Shepard DS Finison LJ Blood pressure reductions: correcting for regression to the mean Prev Med 1983 12 304 317 10.1016/0091-7435(83)90239-6 6878192

[B5] Whitney CW Von Korff M Regression to the mean in treated versus untreated chronic pain Pain 1992 5 281 285 1280801 10.1016/0304-3959(92)90032-7

[B6] Denke MA Frantz ID Responses to a cholesterol-lowering diet: efficacy is greater in hypercholesterolemic subjects even after adjustment for regression to the mean Am J Med 1993 94 626 631 10.1016/0002-9343(93)90215-B 8506889

[B7] Herpin D Demange J Effect of regression to the mean in serial echocardiographic measurements of left ventricular mass: quantification and clinical implications Am J Hypertens 1994 7 824 828 7811441 10.1093/ajh/7.9.824

[B8] Forrow L Calkins DR Allshouse K Horowitz G Delbanco TL Evaluating cholesterol screening: the importance of controlling for regression to the mean Arch Intern Med 1995 155 2177 84 10.1001/archinte.1995.00430200056009 7487239

[B9] Prescott RJ Garraway WM Regression to the mean occurs in measuring peak urinary flow Br J Urol 1995 76 611 613 10.1111/j.1464-410X.1995.tb07787.x 8535681

[B10] Pitts SR Adams RP Emergency department hypertension and regression to the mean Ann Emerg Med 1998 31 214 8 10.1016/S0196-0644(98)70309-9 9472183

[B11] Murakawa Y Yamashita T Ajiki K Sezaki K Omata M Ostensible day-night difference of QT prolongation during long-term treatment with antiarrhythmic drugs: reappraisal of the law of ‘regression to the mean’ J Cardiovasc Pharmacol 1998 32 62 65 10.1097/00005344-199807000-00010 9676722

[B12] Cummings SR Palermo L Browner W Marcus R Wallace R Pearson J Blackwell T Eckert S Black D Monitoring osteoporosis therapy with bone densitometry: misleading changes and regression to the mean JAMA 2000 283 1318 1321 10.1001/jama.283.10.1318 10714731

[B13] Asmar R Safar M Oueneau P Evaluation of the placebo effect and reproducibility of blood pressure measurement in hypertension Am J Hypertens 2001 14 6 Pt 1 546 552 11411734 10.1016/s0895-7061(00)01286-3

[B14] Chapurlat RD Blackwell T Bauer DC Cummings SR Changes in biochemical markers of bone turnover in women treated with Raloxifene: influence of regression to the mean Osteoporosis Int 2001 12 1006 1014 10.1007/s001980170009 11846325

[B15] Takashima Y Sumiya Y Kokaze A Yoshida M Ishikawa M Sekine Y Akamatsu S Magnitude of the regression to the mean within one-year intra-individual changes in serum lipid levels among Japanese male workers J Epidemiol 2001 11 61 69 10.2188/jea.11.61 11388494 PMC11638029

[B16] Krum H Tonkin A Why do phase III trials of promising heart failure drugs often fail? The contribution of ‘regression to the truth’ J Card Fail 2003 9 347 349 10.1054/j.cardfail.2003.08.006 14583896

[B17] Allison DB Loebel AD Lombardo I Romano SJ Siu CO Understanding the relationship between baseline BMI and subsequent weight change in antipsychotic trials: Effect modification or regression to the mean? Psychiatry Res 2009 170 172 176 10.1016/j.psychres.2008.10.007 19897253

[B18] Aaron MT Applegate RA Porter J Thibos LN Schallhorn SC Brunstetter TJ Tanzer DJ Why Preoperative Acuity Predicts Postoperative Acuity in Wavefront-Guided LASIK Optom Vis Sci 2010 87 861 866 10.1097/OPX.0b013e3181f6fb49 20871471 PMC2981088

[B19] Bernstein JA Bernstein E Heeren TC Mechanisms of change in control group drinking in clinical trials of brief alcohol intervention: Implications for bias toward the null Drug Alcohol Rev 2010 29 498 507 10.1111/j.1465-3362.2010.00174.x 20887573

[B20] Taylor CE Jones H Zaregarizi M Cable NT George KP Atkinson G Blood pressure status and post-exercise hypotension: an example of spurious correlation in hypertension research? J Hum Hypertens 2010 24 585 592 10.1038/jhh.2009.112 20054347

[B21] McCall WV D’Agostino R Rosenquist PB Kimball J Boggs N Lasater B Blocker J Dissection of the factors driving the placebo effect in hypnotic treatment of depressed insomniacs Sleep Med 2011 12 557 564 10.1016/j.sleep.2011.03.008 21601519 PMC3110560

[B22] Morton V Torgerson DJ Effect of regression of the mean in decision making in health care BMJ 2003 326 1083 1084 10.1136/bmj.326.7398.1083 12750214 PMC1125994

[B23] Tversky A Kahneman D Judgment under uncertainty: Heuristics and biases Science 1974 185 1124 1131 10.1126/science.185.4157.1124 17835457

[B24] Linden A Use of the total population approach to measure U.S. disease management industry's cost savings: issues and implications Dis Manage Health Outc 2007 15 13 18 10.2165/00115677-200715010-00003

[B25] Galton F Regression towards mediocrity in hereditary stature J Anthropol Inst 1886 15 246 263

[B26] Campbell DT Kenny DA A Primer on Regression Artifacts 1999 New York: Guilford Press

[B27] Gardner MJ Heady JA Some effects of within person variability in epidemiological studies J Chronic Dis 1973 26 781 795 10.1016/0021-9681(73)90013-1

[B28] Johnson WD George VT Effect of regression to the mean in the presence of within-subject variability Stat Med 1991 10 1295 1302 10.1002/sim.4780100812 1925160

[B29] Linden A Adams J Roberts N An assessment of the total population approach for evaluating disease management program effectiveness Dis Manage 2003 6 93 102 10.1089/109350703321908478 14577903

[B30] Ware JE Jr Kosinski M Keller SD A 12-item short-form health survey: Construction of scales and preliminary tests of reliability and validity Med Care 1996 34 220 233 10.1097/00005650-199603000-00003 8628042

[B31] Butterworth S Linden A McClay M Leo MC The effect of motivational interviewing-based health coaching on employees' physical and mental health status J Occup Healt Psych 2006 11 358 365 10.1037/1076-8998.11.4.358 17059299

[B32] Ware JE Losinski M Turner-Bowker DM Gandek B How to Score Version 2 of the SF-12® Health Survey (With a Suppl. Documenting Version 1) 2002 Lincoln, RI: QualityMetric Incorporated

[B33] Davis CE The effect of regression to the mean in epidemiologic and clinical studies Am J Epidemiol 1976 104 493 498 984023 10.1093/oxfordjournals.aje.a112321

[B34] Barnett AG van der Pols JC Dobson AJ Regression to the mean: what it is and how to deal with it Int J Epidemiol 2005 34 215 220 15333621 10.1093/ije/dyh299

[B35] Chesher A Non-normal variation and regression to the mean Stat Methods Med Res 1997 6 147 166 10.1191/096228097672663908 9261913

[B36] Majnu J Abbas FJ Assessing the regression to the mean for non-normal populations via kernel estimators N Am J Med Sci 2010 2 288 292 22558576 PMC3341634

[B37] Lee DS Lemieux T Regression discontinuity designs in econometrics J Econ Lit 2010 48 281 355 10.1257/jel.48.2.281

[B38] Linden A Adams JL Combining the regression-discontinuity design and propensity-score based weighting to improve causal inference in program evaluation J Eval Clin Pract 2012 18 317 325 10.1111/j.1365-2753.2011.01768.x 22304484

[B39] Yudkin PL Stratton IM How to deal with regression to the mean in intervention studies Lancet 1996 347 241 243 10.1016/S0140-6736(96)90410-9 8551887

[B40] Linden A Estimating the effect of regression to the mean in health management programs Dis Manage and Healt Outc 2007 15 7 12 10.2165/00115677-200715010-00002

[B41] Stuart EA Matching methods for causal inference: a review and a look forward Stat Sci 2010 25 1 21 10.1214/09-STS313 20871802 PMC2943670

[B42] Roberts AOH Echternacht G Regression toward the mean and the regression-effect bias New Directions for Testing and Measurement 1980 San Francisco: Jossey-Bass 59 82

[B43] Irwig L Glasziou P Wilson A Macaskill P Estimating an individual’s true cholesterol level and response to intervention JAMA 1991 266 1678 85 10.1001/jama.1991.03470120080037 1886192

[B44] Trochim WMK The Research Methods Knowledge Base 2001 2 Cincinnati: Ohio, Atomic Dog Publishing

[B45] Shadish SR Cook TD Campbell DT Experimental and quasi-experimental designs for generalized causal inference 2002 Boston: Houghton Mifflin

